# Myocardial microvascular patterns in histological sections from patients with and without type 2 diabetes

**DOI:** 10.1371/journal.pone.0354451

**Published:** 2026-07-23

**Authors:** Jaromír Šrámek, Aneta Pierzynová, Vojtěch Melenovský, Tomáš Kučera

**Affiliations:** 1 Institute of Histology and Embryology, The First Faculty of Medicine, Charles University in Prague, Prague, Czech Republic; 2 Department of Cardiology, Institute for Clinical and Experimental Medicine–IKEM, Prague, Czech Republic; Gifu University School of Medicine Graduate School of Medicine: Gifu Daigaku Igakubu Daigakuin Igakukei Kenkyuka, JAPAN

## Abstract

Type 2 diabetes is associated with small vessels dysfunction. This study assessed whether type 2 diabetes is associated with changes in the myocardial capillary pattern in two-dimensional histological sections as an indirect proxy for the three-dimensional network in the myocardium. We used myocardial samples from the left and right ventricles harvested during cardiac surgery (N = 53; 44 without and 9 with type 2 diabetes). We prepared histological sections with CD34-immunostained endothelium. We used cross-sections through the small vessels as points for two methods of point-pattern analysis: Voronoi-based analysis and fractal-based analysis. We were unable to detect differences in the microvascular pattern on myocardial sections of participants with and without type 2 diabetes.

## Introduction

Type 2 diabetes is associated with a wide spectrum of cardiovascular diseases, mainly coronary ischemic disease, cerebrovascular disease, peripheral artery disease, heart failure, and cardiac arrhythmia [1,2].

Pathological changes of the myocardium associated with type 2 diabetes were described in the 1970s as a hypertrophy and fibrosis of the cardiac muscle without significant involvement of coronary arteries. The condition was named diabetic cardiomyopathy. The authors assumed that the pathological changes are related to the diabetic microangiopathy [3]. Diabetic cardiomyopathy leads to the left ventricular hypertrophy, diastolic dysfunction, systolic dysfunction and impaired contractile reserve. Myocardial fibrosis is both interstitial and perivascular and can be responsible for the worsening of mechanical properties of the heart [4].

Pathogenesis of diabetic cardiomyopathy is thought to be initiated by elevated glucose levels. At the level of the extracellular matrix, production of advanced glycation end-products (AGEs) is increased. AGEs directly affect mechanical properties of the tissue [5]; they are also recognized by pro-inflammatory receptors of immune cells [6]. At the level of cardiac muscle cells, insulin resistance is developing. Cardiac muscle cell metabolism is impaired. The impairment includes higher uptake of fatty acids, mitochondrial stress and endoplasmic reticulum stress [7]. Impaired metabolism may lead not only to the stress-related paracrine signaling but also to cell death [8]. The common denominator of both extracellular and intracellular pathological processes is that both processes can cause chronic stimulation of the immune system. Long-lasting activation of the immune cells in the myocardium has the potential to affect the tissue homeostasis [9].

Vascular complications of type 2 diabetes are common at all calibers, from the aorta to the capillaries [10]. Diabetic microangiopathy affects mainly the retina, the kidney, and the nerve tissue, but other organs, including the heart, can be affected as well [11]. Imaging-based studies have shown impaired perfusion of the myocardium associated with type 2 diabetes [12,13].

Several morphological abnormalities of microvessels in diabetic hearts have been described. Fischer et al. [14] described thickening of the capillary basement membrane associated with diabetes. Thompson [15] described decreased capillary density and diameter of capillaries associated with diabetes in an animal model of diabetes. Gherasim et al. [16] described decreased microvascular density associated with diabetes in samples obtained from autopsies. In contrast, Ledet [17] found no evidence of changes in the microvascular density associated with diabetes in samples obtained from autopsies of young men with type 1 diabetes. Campbell et al. [18] did not reveal a disturbance of microvascular structure associated with type 2 diabetes using measures as follows: capillary length density, diffusion radius, and arteriolar dimension.

Both chronic inflammation and structural remodeling of the myocardium cause structural changes in the microvascular network. Previous analyses focused on structural changes of the microvascular network [15–18] have been inconclusive. We therefore hypothesized that any such changes may be subtle and may be reflected in the spatial arrangement rather than in mean values. Therefore, we used methods based on the point-pattern analysis, which are more informative than simple measures such as microvascular density or capillary length density.

## Materials and methods

### Patients and histological processing

For the analysis, samples of myocardium were harvested from explanted hearts during transplantation surgery. This was a secondary analysis of samples collected in previous studies. [19,20]. Inclusion criteria for the previous publications were indication for heart transplantation for advanced heart failure of any underlying etiology and informed consent of the patient with further analysis of the explanted heart. The exclusion criterion was age below 15 years. The explanted hearts underwent mandatory examination at the department of pathology; pathologists harvested samples for the histological analysis.

The number of samples used in this work is lower than the sum of samples in previous studies due to the limited volume of remaining biological material that prevented reuse of the material for the analysis in some cases. Only samples with an appropriate volume of remaining tissue were included. For the study, we used samples from 53 patients: 44 without type 2 diabetes and 9 with type 2 diabetes.

The subsequent analysis was conducted in accordance with the approval obtained for the previous studies which were approved by the Ethics Committee of the Institute for Clinical and Experimental Medicine and Thomayer Hospital with Multi-center Competence, decision G-16-06-28, and was performed in accordance with the guidelines proposed in the Declaration of Helsinki (2000) of the World Medical Association. Written informed consent permitted subsequent analysis of the samples. For this study, we were completely blinded regarding personal or clinical data except for the presence or absence of type 2 diabetes.

Samples of myocardium were fixed in 4% formaldehyde, embedded in paraffin, and cut into 6-micrometer-thick sections. We used immunostaining for CD34 (Monoclonal Mouse Anti-Human Antibody, clone QBEnd 10, DakoCytomation, Glostrup, Denmark) for highlighting capillaries.

The microscope Leica DMLB (Leica Microsystems GmbH, Wetzlar, Germany) with 40 times magnification of the objective was used to capture images. At least 10 non-overlapping images were obtained. For analysis of microvascular pattern, areas with the plane of section perpendicular to the long axis of cardiomyocytes were used. Each vessel with a diameter smaller than 10 micrometers was assumed to be a microvessel and was labeled manually. The value 10 micrometers is often used as a conventional border for capillaries [21]. To mitigate interobserver variability, only one author (A.P.) performed this analysis. A.P. is a scientist experienced with this type of analysis. During this step, A.P. was blinded to information about the sample. For the analysis, non-overlapping images were systematically acquired from across the entire sample area. The centerpoint of each vessel was recorded as relative coordinates in the image.

### Voronoi-based analysis

There are numerous stereological methods of analysis of the microvascular network in histological sections. Commonly used parameters are the volume fraction of capillaries, the surface of endothelium, the length of capillaries, and especially the number of capillaries per surface unit, often called the microvascular density [22]. Voronoi-based approach to the point pattern is a generalization of measures like the microvascular density; therefore, those measures were not calculated.

Voronoi tessellation of any plane, or in general, any metric space regarding a set of n points called centroids, is a segmentation of the plane into n convex polygons called Voronoi cells. Voronoi cells have the property that each point inside a given Voronoi cell is closer to the centroid in the Voronoi cell than to other centroids.

In the two-dimensional case, Voronoi cells are polygons surrounding centroids. If the position of centroids can be assumed as a result of a Poisson process, i.e., the position of each centroid is independent on the position of other points, statistical properties of areas of Voronoi cells can be fitted by generalized gamma distribution. Moreover, standardizing areas of Voronoi cells by dividing by the mean area of Voronoi cells leads to fixing all three parameters of generalized gamma distribution [23].

The assumption about the Poisson process behind the capillary pattern is not plausible for biological reasons. Capillaries do not seem to be distributed homogeneously in the plane of section; rather, they tend to cluster. A simple explanation of this is natural branching of the capillary network. Therefore, another statistical model is necessary to describe the statistical behavior of areas of Voronoi cells. Several models have been proposed without the analytical proof of correctness; they are based rather on their empirical usefulness. Usually, the Pareto distribution, lognormal distribution, exponential distribution, and gamma distribution are used [24]. Šrámek et al. [25] demonstrated that lognormal distribution is the best choice for analysis of the microvascular pattern in the myocardium.

Parameters of the distribution (LOGMEAN and LOGSD) allow calculation of a conventional parameter microvascular density (MVD) as follows:


$$\begin{array}{c} MVD=\exp\left(-LOGMEAN-\frac{LOGSD^{2}}{2}\right) \end{array}$$ (1)

Image processing was performed by a script written in the Octave language (ver. 8.4.0). Main segmentation was carried out using the function voronoin. Only complete cells were used for further analysis, i.e., unbounded cells were excluded. Areas of cells served for calculation of parameters LOGMEAN and LOGSD of the lognormal distribution [26].

### Fractal-based analysis

A pattern composed of a finite number of points has the fractal dimension equal to zero. Algorithms used for estimation of the fractal dimension can give a non-integer result as an artifact of the chosen method. This number can be related to the complexity of the analyzed shape on the digital picture [27].

For estimation of the fractal dimension (FD), we used an algorithm based on Dubuc’s approach to the fractal dimension [28]. Briefly, we replaced points by discs of bigger and bigger diameter. In each step, the diameter of covering discs r and covered area A were recorded. According to theory, these data should fit the line in the log-log plot:


$$\begin{array}{c} \ln\frac{A}{r^{2}}=-\text{FD}\cdot \ln(r)+const, \end{array}$$ (2)

The fractal dimension is estimated as a slope of the line. For the point pattern, the log-log plot appears to be rather a ramp function. We reduced the number of parameters of the fitting function from [28]. Therefore, we have used the function as follows:


$$\begin{array}{c} f(x;A,B,C)=A-B\ln\left(1+e^{Cx}\right) \end{array}$$ (3)

Parameters A, B, and C in equation 3 are parameters that have to be estimated and used for estimation of the fractal dimension by fitting the log-log plot by the function nonlin_curvefit from the package optim in the Octave language (ver. 8.4.0).

Fractal dimension even of non-fractal pattern can be estimated from the above mentioned parameters using the oblique asymptote of the function 3, the result can be expressed using parameters B and C [28]:


$$\begin{array}{c} \text{FD}=-BC \end{array}$$ (4)

### Statistical analysis

Statistical analysis was performed in the R language (ver. 4.3.3). For visualization of the results, the library ggplot2 was used. For comparison between two groups, the Mann-Whitney test was used.

## Results

For the main analysis, we included samples from 53 participants. We included only those participants with a sufficient amount of material from both the left and right ventricles. Of those 53 participants, 9 participants had type 2 diabetes and 44 participants did not have type 2 diabetes. The age of participants was between 16 and 68 years; the mean age was 51 years. The mean age of participants with type 2 diabetes was 57 years, and the mean age of participants without type 2 diabetes was 50 years. Only 9 (17%) participants were female, the majority of participants were male (83%). All participants with type 2 diabetes were male, the majority of participants without type 2 diabetes were male (80%).

A brief summary of calculated descriptors of the capillary pattern is provided in Table 1.

**Table 1:** Summary of Voronoi-based descriptors and fractal-based descriptors.

	Left ventricle	Right ventricle
LOGMEAN	6.386±0.259	6.379±0.222
LOGSD	0.3960±0.0432	0.4064±0.0543
MVD	1611±450	1602±352
FD	0.6208±0.0292	0.6189±0.0288

Summary of Voronoi-based descriptors and fractal-based descriptors of the capillary pattern of all 53 samples. Values have the structure mean ± standard deviation. Note that the dimension of the physical pixels was included in the analysis; therefore, the dimension of the values of LOGMEAN and LOGSD is related to a square micrometer. FD is dimensionless. Conventional parameter microvascular density (MVD) was calculated from LOGMEAN and LOGSD using the formula 1, its dimension is capillaries per square millimeter.

**Fig 1:**
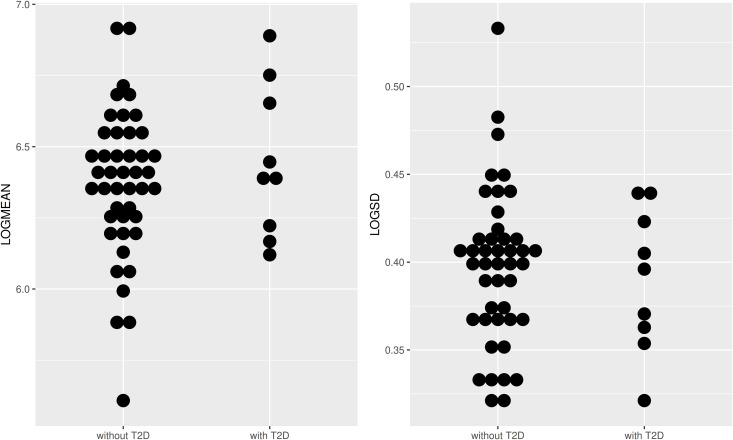
Voronoi-based descriptors of the left ventricle. Box plots of LOGMEAN (left) and LOGSD (right) of the left ventricle according to the presence or absence of type 2 diabetes.

**Fig 2:**
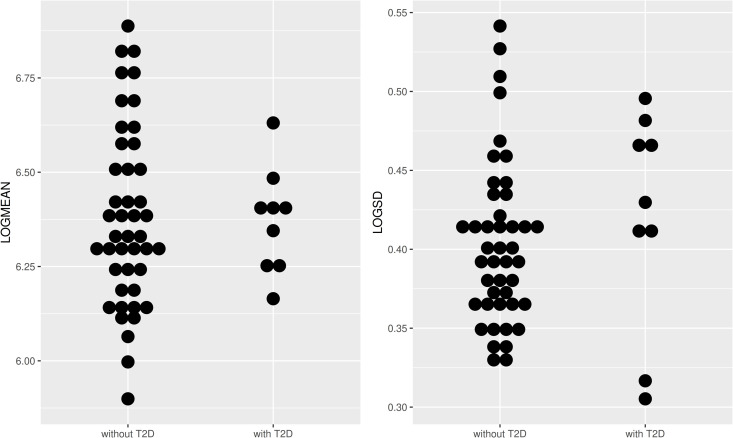
Voronoi-based descriptors of the right ventricle. Box plots of LOGMEAN (left) and LOGSD (right) of the right ventricle according to the presence or absence of type 2 diabetes.

Values of LOGMEAN and LOGSD grouped regarding the presence of type 2 diabetes are shown in Fig 1 (left ventricle) and in Fig 2 (right ventricle). We performed the Mann-Whitney test for comparison of subgroups with and without type 2 diabetes in the left and right ventricles. In the left ventricle, the p-values were 0.6835 for LOGMEAN and 0.7356 for LOGSD. In the right ventricle, the p-values were 0.8614 for LOGMEAN and 0.2758 for LOGSD. Therefore, we were unable to detect a difference.

**Fig 3:**
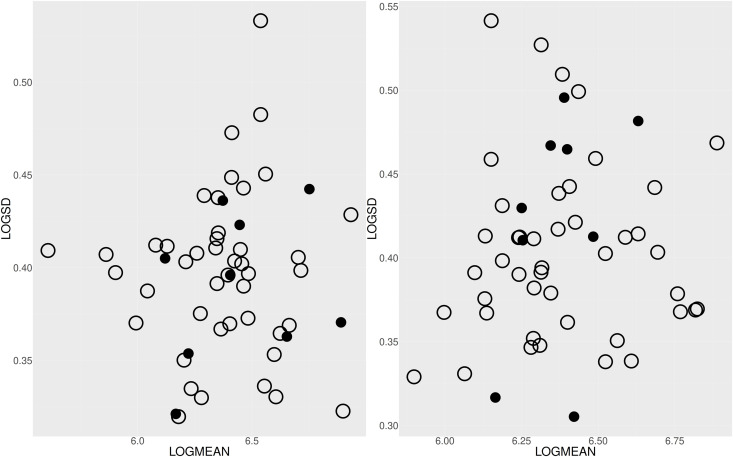
Scatter plots of LOGMEANs and LOGSDs. Scatter plots of LOGMEAN and LOGSD of the left (left side) and right (right side) ventricle. Empty circle denotes a subject without type 2 diabetes, smaller filled disc denotes a subject with type 2 diabetes.

LOGMEAN and LOGSD contain part of one piece of information; their combination may be helpful. The scatter plot for subjects with and without type 2 diabetes is in Fig 3. Visual inspection of the pattern in figure 3 does not support the hypothesis that LOGMEAN and LOGSD together can distinguish samples with and without type 2 diabetes.

**Fig 4:**
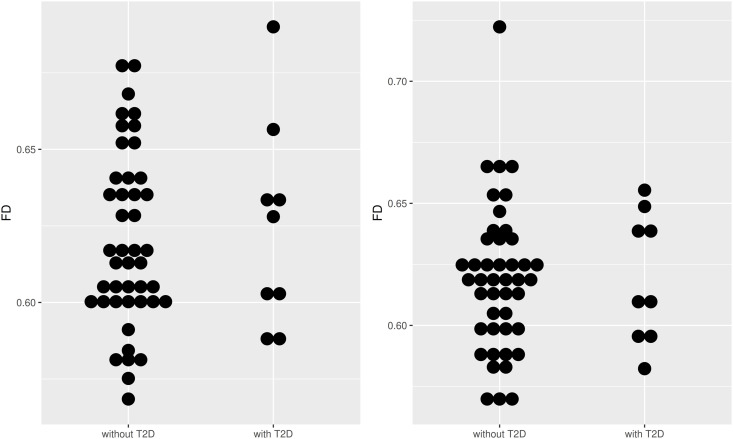
Fractal-based descriptors. Box plots of values of FD in the left (left pair) and right (right pair) ventricle regarding presence or absence of type 2 diabetes.

Values of FD grouped according to the presence or absence of type 2 diabetes are in Fig 4. We performed the Mann-Whitney test for comparison of subgroups with and without type 2 diabetes. The p-values were 0.8431 and 0.9166 for the left and right ventricles, respectively. Therefore, we were unable to detect a difference.

## Discussion

We demonstrated two approaches to the description of the microvascular pattern on the histological section as an indirect proxy for the three-dimensional capillary network. Both used approaches have potential advantages. In comparison with the widely used microvascular density, it also considers irregularities of the pattern.

Voronoi-based descriptors can be transformed into parameters with more direct biological interpretation, such as microvascular density [25]. Fractal-based descriptors are scale-independent not only in theory but also in applications [27]. This property can be kept even if the approach is used for analysis of a non-fractal pattern [28]. This class of descriptors allows us to describe irregularities or complexity of the pattern [27]. Fractal-based descriptors also have one advantage. Because they are scale-independent [28], they are by nature insensitive to shrinkage of tissues during histological processing [29].

The main goal of this work was to test the hypothesis that the three-dimensional structure of the capillary network in the myocardium is affected by type 2 diabetes. We tested the hypothesis using conventional two-dimensional sections which were an indirect proxy of three-dimensional structure. Both used methods are primarily methods of analysis of a point pattern. Voronoi tessellation centered on capillaries was used for estimation of the area of oxygen supply in the skeletal muscle. Even in the skeletal muscle, this approach leads to an inappropriate result under more complex circumstances [30]. The fractal dimension of a 2D section is related to the fractal dimension of a 3D object, but the association drops for non-isotropic biological structures [31]. Taken together, those results limit biological interpretation of detected differences in the sense of direct analysis of biological importance of the differences. However, the absence of detectable difference is consistent with the possibility that structural coronary microvascular dysfunction [32] may play a role in the pathophysiology of diabetic cardiomyopathy rather than remodeling of the structure of the microvascular network.

We were unable to detect a difference between the patterns of capillary structure in myocardium associated with and without type 2 diabetes. This observation is consistent with previously published studies [17] and [18].

Focusing on the biology of capillaries, they show not only morphological disturbances [12] but also profound functional changes [29,33,34]. In the myocardium, morphological remodeling associated with diabetes was described [35]. The remodeling includes overproduction of connective tissue [33], cardiac muscle remodeling [4], and changes of cardiac muscle cells [36]. Those disturbances can lead to disturbances of the three-dimensional structure of the capillary network. Therefore, our negative finding needs explanation.

First, pathological processes can act on capillaries in exactly opposite directions and intensities; therefore, they are balanced. This scenario appears to be unstable, and one can expect that in association with other pathologies, this putative balance will be broken easily. Despite the low probability of the scenario in general, we cannot omit it because our sample consists of older patients with heart failure, often with additional comorbidities. The limited number of samples in our study also increases the risk of this kind of bias.

Second, the density of capillaries in the myocardium is constant or even nearly constant even under pathological conditions. This explanation is consistent with observations that, e.g., atrial fibrillation is also not associated with detectable disturbances of the microvascular density [37,38].

There were published positive results, which are conflicting with our observation. Thompson et al. [15] detected the association. But in contrast to natural disease, they used an animal model with artificially induced disease and with a long period without an appropriate level of glucose. Myocardial samples in the study are rather a model of advanced stages, whereas the severity of diabetes of our participants is unknown.

Gherasim et al. [16] showed a decline in the density of arterioles in myocardium associated with type 2 diabetes. We focused on capillaries; therefore, their result is only indirectly related to our result. If the density of arterioles decreases but the density of capillaries is not changed, branching of capillaries should be more pronounced. This property can be missed by analysis of the density of vessels only but should be detected by our approach as the existence of crowding of capillaries, i.e., more Voronoi cells with small area. We were unable to confirm the explanation.

Other studies [17,18] were unable to detect structural changes in the pattern of the capillary network in histological section associated with type 2 diabetes, which is in concordance with our observation.

Taken together, we are convinced that the disease stage and glycaemic control may be important. Both published negative results [17,18] and our negative result were obtained from a general clinical population with known type 2 diabetes, without information about advanced issues related to poor glycaemic control. Moreover, our samples were from explanted hearts obtained during transplantation surgery; in this situation, advanced type 2 diabetes with complications is improbable because type 2 diabetes with complications is the relative contraindication of the transplantation [39]. In contrast, study [15] was performed on model animals, but with prolonged poor compensation of induced diabetes. It suggests that remodeling can be the result of the long-lasting influence of inappropriate compensation for diabetes.

A substantial limitation of our study is the small sample size, especially the number of participants with type 2 diabetes, which is low. Therefore, a negative result is associated with increased risk of type II error, and the statistical power of the result is limited. This negative finding should be interpreted mainly in the context of similar negative findings in other studies. Moreover, due to the low statistical power, especially low or moderate differences can be missed.

The second limitation is that we were blinded to the full record of the participants. Even if information on important confounders such as age, sex and comorbidities were available, the small number of participants with type 2 diabetes would limit multivariable analysis. Because common serious conditions leading to the heart transplantation can affect the structure of myocardium and structure of capillary network, there is a high risk that additional factors affecting the structure of the myocardium are not balanced between groups with and without type 2 diabetes. Therefore, our result should be interpreted with caution.

In the analysis, only one person processed the images to prevent interobserver variability. This approach minimized interobserver variability. However, the data may still be affected by systematic observer bias, which may limit comparability with other datasets.

## Conclusion

We compared microvascular (capillary) patterns in histological specimens of myocardium harvested during cardiac surgery using two different techniques. In this small cohort, we were unable to detect differences between patients with type 2 diabetes and without type 2 diabetes. Because the study had limited statistical power, lacked detailed clinical information, and relied on two-dimensional sections as an indirect proxy for a three-dimensional capillary network, the findings should be interpreted cautiously. They suggest that type 2 diabetes in this cohort is not associated with detectable changes in the myocardial capillary pattern, whereas the effects of poorly controlled type 2 diabetes with end-organ complications remain unresolved.

## Supporting information

S1 Data T1-raw data. (CSV)

S2 Data T2-clinical data. (CSV)
